# Defining tissue proteomes by systematic literature review

**DOI:** 10.1038/s41598-017-18699-8

**Published:** 2018-01-11

**Authors:** Sarah A. Hibbert, Matiss Ozols, Christopher E. M. Griffiths, Rachel E. B. Watson, Mike Bell, Michael J. Sherratt

**Affiliations:** 1 Division of Cell Matrix Biology & Regenerative Medicine, The University of Manchester, Manchester, UK; 2 Centre for Dermatology Research, Faculty of Biology, Medicine and Health, The University of Manchester, Manchester, UK; 3 Salford Royal NHS Foundation Trust, Manchester Academic Health Science Centre, Manchester, UK; 4 NIHR Manchester Biomedical Research Centre, Central Manchester University Hospitals NHS Foundation Trust, Manchester Academic Health Science Centre, Manchester, UK; 5 Walgreens Boots Alliance, Thane Road, Nottingham, UK

## Abstract

Defining protein composition is a key step in understanding the function of both healthy and diseased biological systems. There is currently little consensus between existing published proteomes in tissues such as the aorta, cartilage and organs such as skin. Lack of agreement as to both the number and identity of proteins may be due to issues in protein extraction, sensitivity/specificity of detection and the use of disparate tissue/cell sources. Here, we developed a method combining bioinformatics and systematic review to screen >32M articles from the Web of Science for evidence of proteins in healthy human skin. The resulting Manchester Proteome (www.manchesterproteome.manchester.ac.uk) collates existing evidence which characterises 2,948 skin proteins, 437 unique to our database and 2011 evidenced by both mass spectrometry and immune-based techniques. This approach circumvents the limitations of individual proteomics studies and can be applied to other species, organs, cells or disease-states. Accurate tissue proteomes will aid development of engineered constructs and offer insight into disease treatments by highlighting differences in proteomic composition.

## Introduction

The past decade has offered extensive advancements in numerous “omics” techniques allowing a much more comprehensive overview of organisms and the onset of pathologies. Ideally both genomics and proteomics would advance sufficiently to offer personalised medicine, by characterising disease phenotypes and informing pharmacogenomics for the prevention and/or treatment of diseases^1^. However, whilst genomic advancements now allow the entire human genome to be rapidly sequenced (at a fraction of the cost a decade ago^2^) defining tissue specific proteomes remains a much more challenging task. Whilst genomics and transcriptomics provide a ‘snap-shot’ of the genes and mRNA at a specific time-point, this does not provide an accurate representation of the constitutive proteins within a tissue, particularly long-lived proteins deposited in early development.

Whilst there is an abundance of data that list entire species proteomes, upon investigation it has become apparent that data on tissue specific proteomes is severely lacking. Currently, information on the identity of tissue/organ specific proteomes can only be found in two databases; the Human Protein Atlas (HPA), which relies heavily on transcriptomics and does not organise its data according to proteomic evidence^3^ and PaxDB^4^, which collates mass spectrometry (MS) data where the primary data source and complete experimental detail cannot be accessed. We searched for data on cartilage, aorta and skin proteomes, for both cartilage and aorta only a few MS papers were found all containing differing numbers and identities of constituent proteins and neither had entries on HPA or PaxDB. HPA, PaxDB and 14 experimental proteomes, primarily MS studies, report on the human skin proteome, whilst these resources each have their own merits, when comparing both the number and identity of proteins listed we found considerable disparities (Table 1). The major problems when comparing these proteomes were the differences in protein identification techniques (micro-array, immunohistochemistry [IHC] and MS data), differing sample numbers, anatomical sites, disease states and sometimes the inclusion of cell-line data. It was also striking that a large number of proteins, in particular extracellular matrix (ECM) components, which are vital to skin structure and/or function, were absent from these proteomes. Hence raising the question, how do we define an accurate tissue proteome?

**Table 1:** A comparison of the existing skin proteomes and their corresponding number of proteins, methods of protein evidence and skin source (if available).

Data Source	Reference	Protein #	Evidence	Tissue Source
PaxDB	http://pax-db.org	4139	MS	Unknown
Bliss 2016	10.1186/s12575-016-0045-y	1175	MS	Digit
Raymon 2008	10.1074/mcp.M700334-MCP200	825	MS	Breast
Protein Atlas	http://www.proteinatlas.org	459	mRNA/IHC	Unknown
Parkinson 2014	10.1371/journal.pone.0097772	294	MS	Forearm
Mikesh 2014	10.1016/j.jprot.2013.03.019	147	MS	Leg, Breast
Broccardo 2011	10.1016/j.jaci.2010.10.033	35	MS	Arm
Gromov 2003	10.1074/mcp.M200051-MCP200	35	MS	Forearm
Ong 2010	10.1111/j.1365-2133.2010.09660.x	23	MS	Breast, groin, forearm, thigh, neck abdomen,
da Silva 2015	10.1038/jid.2014.396	22	MS	Unknown
Laimer 2010	10.1111/j.1600-0625.2010.01144.x	19	MS	Foreskin
Bakondi 2011	10.1016/j.freeradbiomed.2010.10.700	17	IHC	Leg
Pollins 2007	10.1016/j.jss.2007.01.001	12	MS	Breast
Kim 2012	10.1186/1477-5956-10-50	10	MS	Forehead, face
Nakamura 2010	10.1111/j.1745-7270.2008.00484.x	10	MS	Unknown
Vainionpää 2007	10.1007/s00441-006-0366-2	10	HC	Unknown

Given our interest in skin, establishing an accurate reference proteome would be an invaluable biological resource for: defining the baseline protein composition of healthy tissue; understanding remodelling associated with ageing and disease phenotypes and; guiding the engineering of human skin constructs. In this study we aimed to: characterise the scale of and consensus between existing skin proteomes; develop a novel method for defining tissue proteomes using existing peer-reviewed literature and; to apply this method to produce a new proteome for an exemplar organ (healthy human skin).

## Results

### Characterisation of existing Skin Proteomes

To assess the consensus amongst skin proteome data we compiled a list of existing proteomics studies and resources (Table 1). There were clear disparities between existing proteomes with regards to the number of proteins, the validation techniques and the tissue sources used. To further assess these resources we compared data from the two proteomics databases HPA (skin enriched) and PaxDB, in addition to the largest MS study we could find in published literature Bliss 2016, classifying each proteome according to protein number, identity and subcellular location^5^.

**Figure 1:**
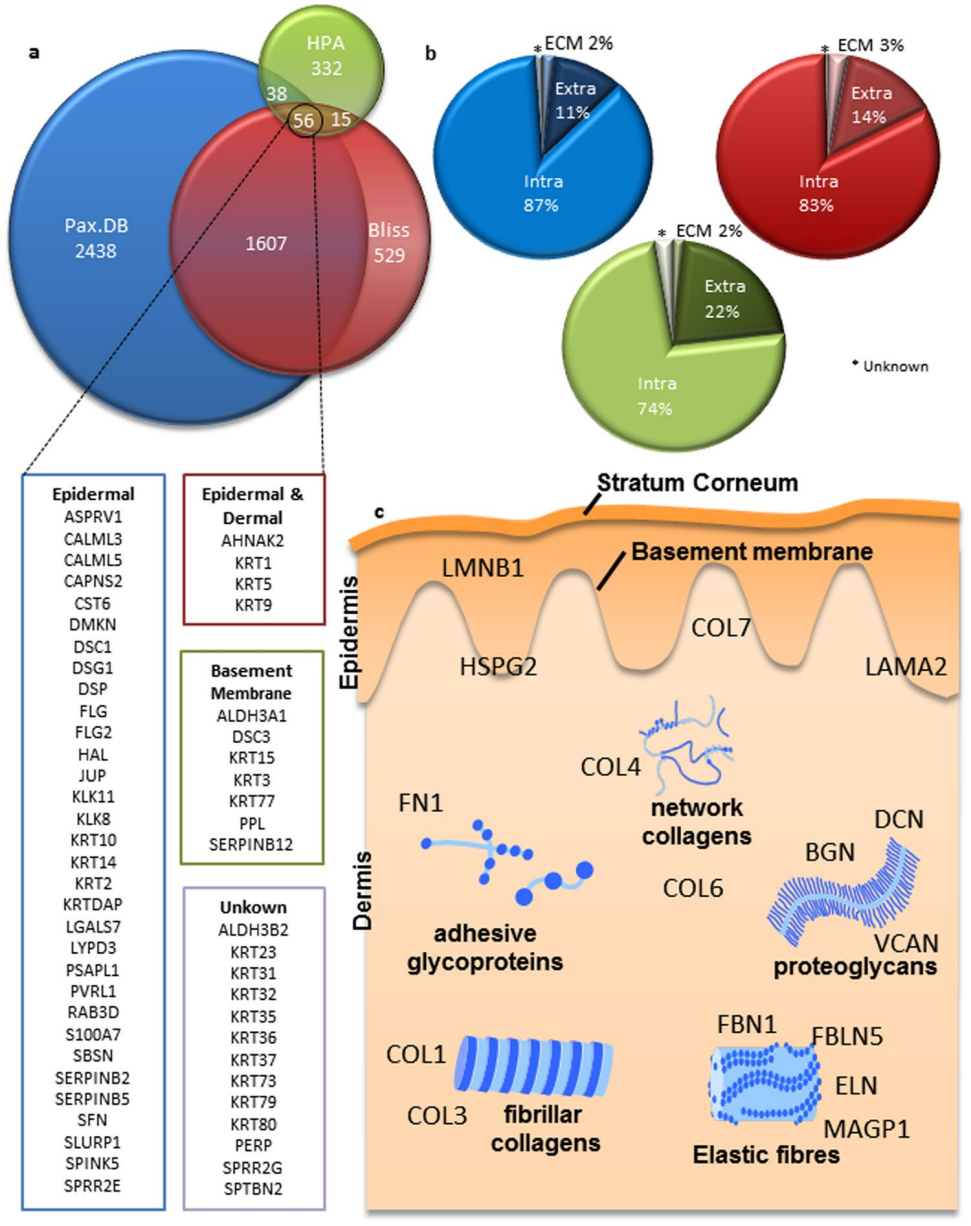
Characterisation of existing Skin Proteomes. To assess the existing proteomics data available we downloaded the contents of two databases, Pax.DB and Human Protein Atlas (HPA) in addition to the largest single skin proteomics paper Bliss 2016, below is a list of the 56 common proteins to all of the analysed proteomes. (**a**) Shows the number of proteins in each database and any consensus between the data. (**b**) Using GO terms the corresponding data was assessed for the subcellular location of each protein listed in the databases; here we show there is a general consensus with regards to subcellular location of proteins but that the absolute values are disparate between the databases. What is clear from the analysis is the obvious lack of proteins commonly associated with the skin, (**c**) here we show some key examples of proteins not listed in the 56 consensus proteins and their locations within the skin.

Our findings demonstrate disparity in not only the total number of proteins in each database (PaxDB = 4139, HPA = 441 and Bliss 2207), but also the identity of these proteins (Fig. 1a). The most unexpected result of this analysis is that only 56 proteins were identified as common to all three databases and neither collagen I nor elastin, the two most abundant skin proteins, were present in this list. The lack of consensus between proteomes was also highlighted when comparing the relative percentages of proteins in each subcellular location (Fig. 1b), where the only agreement between the three databases was that the ECM category contained the fewest number of proteins. An additional resource called the Matrisome. DB (http://matrisomeproject.mit.edu/) focuses on the specific identification of ECM proteins in tissues. When using the ECM proteins identified in Matrisome. DB to highlight skin specific ECM components in PaxDB, HPA and Bliss, only 26.2%, 2.6% and 23.6% coverage respectively were found in each proteome^6^.

Whilst each of these proteomes has their own merits, the disparity in both protein number and identity highlights the difficulties in defining an accurate skin proteome. This lack of consensus between each proteome may be due to difference in protein identification techniques and tissue sources. The categorisation of HPA proteins in organs or tissues for example is reliant on RNA expression, which can indicate protein expression^7–9^ but is much more reflective of protein turnover than absolute abundance or historical protein synthesis^10,11^. This issue is particularly important in ECM-rich tissues (such as the dermis) where, unlike cellular proteins whose half-lives are a maximum of days^12^, protein half-life can be as long as 80 years e.g. elastin^13,14^. Using lung as an example, HPA highlights 183 lung enriched proteins however; elastin is not included in this list. A smaller percentage of the proteins from each tissue specific proteome on HPA were also validated by IHC, which was not always on healthy tissue or a representative description of the staining. Whilst immune-based assays can provide information on protein presence, tissue location and to a lesser extent abundance, there are numerous problems with using these assays as an investigative tool, including: cost; time; optimisation and; antibody availability/efficacy^15–17^.

The remaining skin proteome databases rely on MS as a proteomics discovery tool. However, MS protein analysis alone is also prone to methodological problems. Common proteomics methodologies use what is now termed a ‘bottom up’ approach where a sample is digested into peptide fragments and analysed based on peptide hits from a protein database^18^. The major problem with the ‘bottom up’ approach is the initial sample preparation, where protein extraction is key^19^, particularly in tissues rich in ECM where proteins are large, complex structures that are often insoluble. However, developing technologies now also allow for a ‘top down’ approach where whole protein investigation is possible^20^, given the current limitations in molecular weight (limited to proteins less than 105 KDa) it is not suitable for complete proteomic analysis^21^. For both approaches, establishing the false positive discovery rate is key for accurate analysis of MS data; this technique is widely used in both genomics and transcriptomics but poses a problem in proteomics given the often limited sample sizes^22,23^.

### The Manchester Skin Proteome

**Figure 2:**
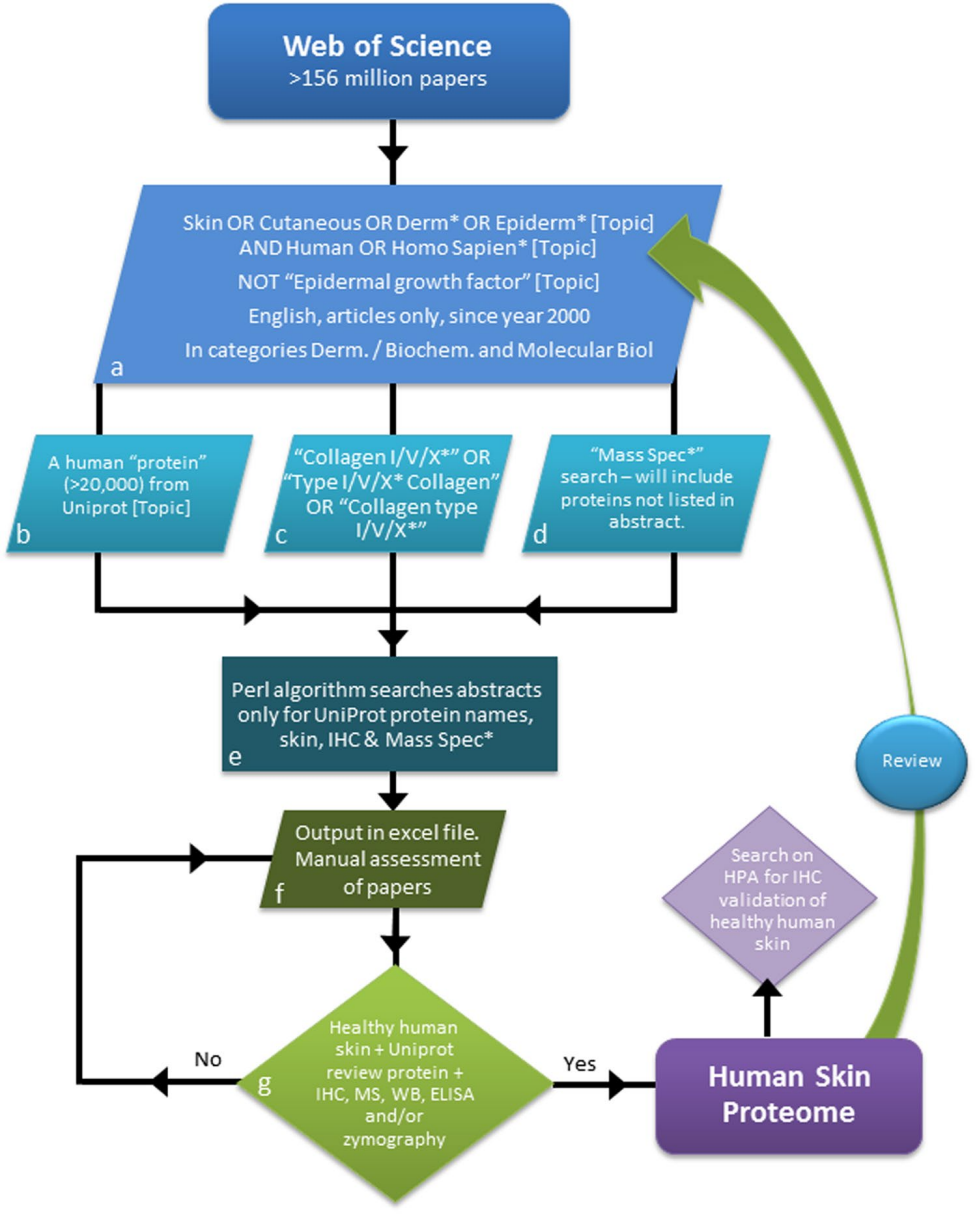
Developing a bioinformatic and systematic literature review for defining proteomes. The entire body of Web of Science literature was searched. A series of terms were used to select a smaller population of publications of interest and the abstract text for each paper was then downloaded (**a**–**d**). A Perl algorithm was developed to search the downloaded abstract text for: at least one protein name as stated in Uniprot, “skin,” “Immunohistochem*”, and/or “Mass Spec*” (**e**) and created an Excel file with the list of DOIs with the contained terms (**f**). The papers in the Excel list were then manually reviewed for evidence (IHC, MS, ELISA, WB or zymography) of the protein in healthy human skin (**g**). Proteins were added to the database with captured data on: protein name, accession number, and gene name (from Uniprot) and; from target articles: skin sample information (age, sex, and anatomical site), localisation within the tissue and any associated pathologies.

To circumvent the limitations in using any single technique or study to provide accurate whole tissue proteomics data, we propose to make use of the largest, most comprehensive source of information on tissue composition, peer-reviewed literature. The key issue with this approach is locating this information within the text of over 100 million peer-reviewed research articles. Therefore, we adapted the principles of a systematic literature review^24^ augmented with bioinformatics text mining techniques (summarised in Fig. 2), to produce a proteomic database that collates data from numerous disparate sources.

In excess of 5,000 papers were manually assessed for proteins in healthy human skin and 343 papers fall under the inclusion criteria, to yield 1,817 proteins in the initial search for the MSP. Following our annual update an additional 744 papers were manually reviewed and 28 papers added to the database. Collectively these publications contained experimental evidence for the presence of 2,948 proteins in healthy human skin.

**Figure 3:**
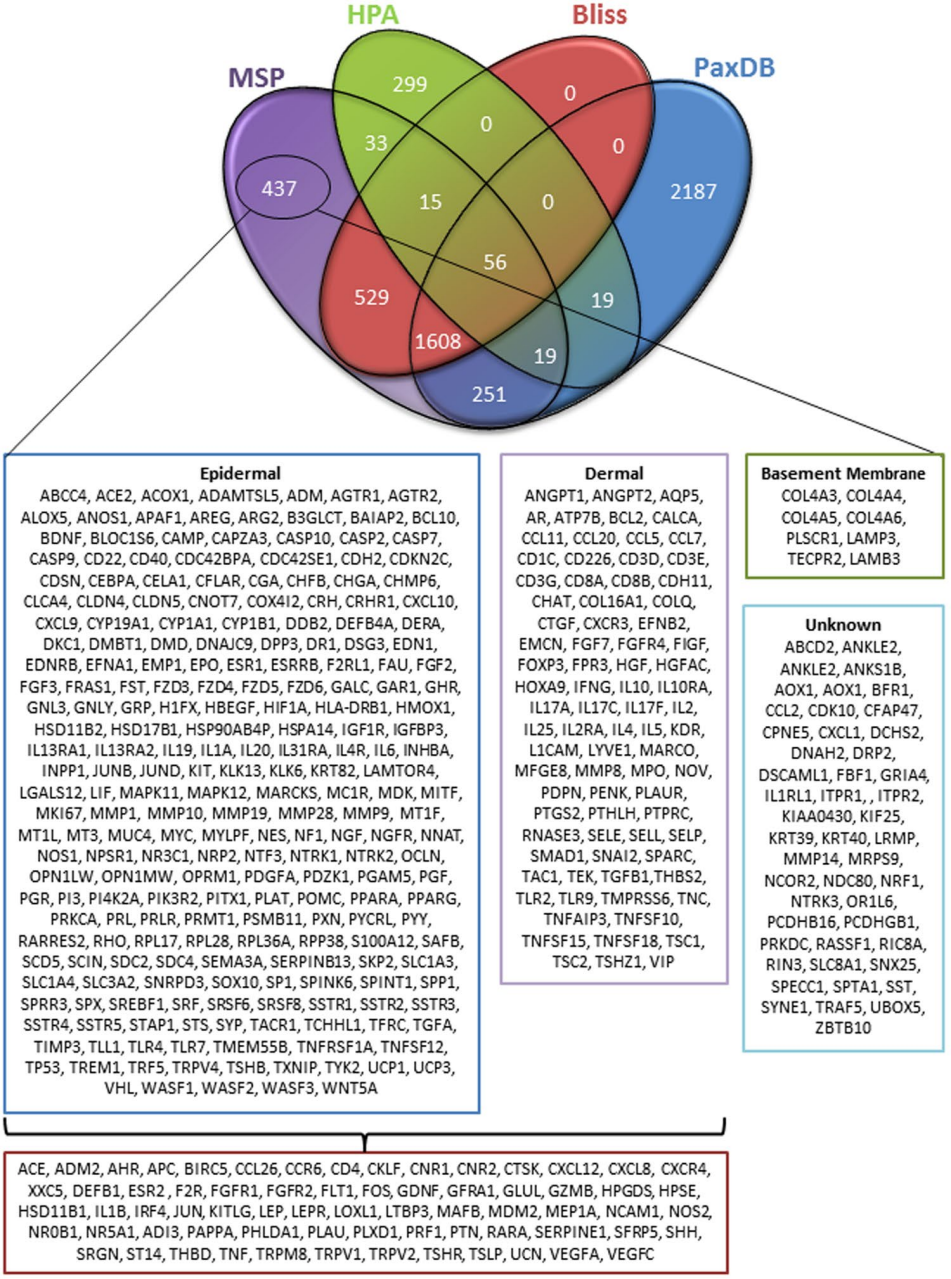
The consensus of The Manchester Skin Proteome with existing databases. To assay the consensus of our proteome compared to the existing proteomes we used an R programming script to generate the four way comparison Venn diagram. From this analysis we can see that 437 proteins in our proteome are unique to our database and below is the list of these proteins and their tissue location if the information was available.

**Figure 4:**
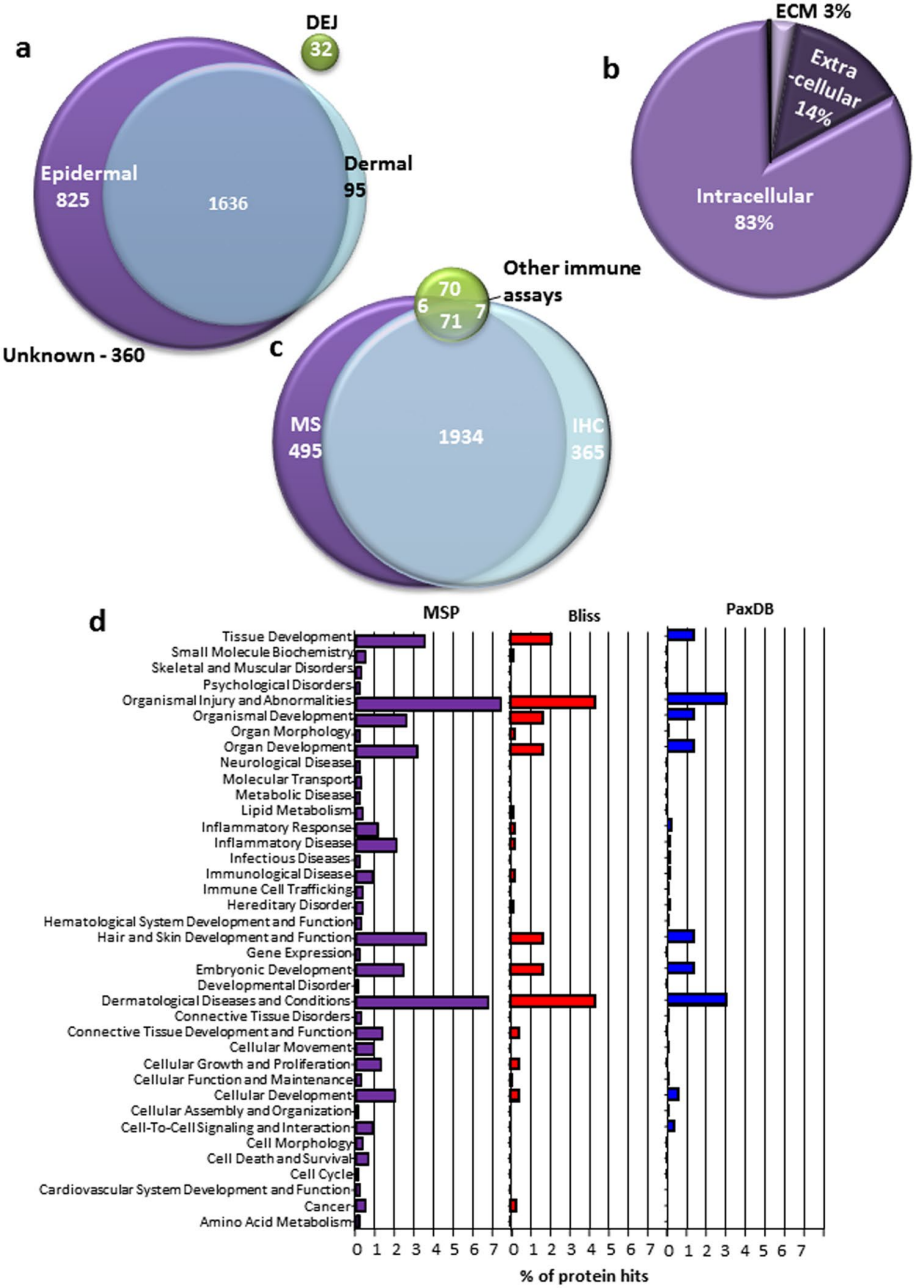
Analysis of The Manchester Skin Proteome. We analysed the data present in our proteome of 2948 proteins. (**a**) Shows the tissue location of the proteins evidenced where information was available within the manuscript. The unknown proteins have been evidenced using only Mass Spectrometry techniques. (**b**) Using GO terminology the subcellular location of each of our proteins was collected, this data is very similar to the patterns observed in the other proteomes where the fewest number of proteins are found in the ECM. (**c**) Finally we assess the techniques used to evidence protein presence, Mass Spectrometry has evidenced the highest number of proteins, and crucially we have duel evidenced 2011 proteins by both Mass Spectrometry and Immuno-based techniques. (**d**) Ingenuity was used to count the number of gene hits in each of the top level Kegg pathways for the MSP, Bliss and PaxDB. We standardised these values as a percentage of hits based on the total number of proteins in each database. Whilst the pattern of observed hits in each of the functional groups in similar across each proteome, the MSP contains more hits than any other database showing a more complete profile of tissue function.

Eighty five percent of the proteins characterised in our MSP were also identified in HPA, PaxDB and/or Bliss (Fig. 3). Crucially the MSP characterised 437 proteins unique to our database and of these, 78% were evidenced using immune-based techniques only. In addition 88% of proteins in our database had tissue location information associated with them; when MS evidence of whole human skin was evaluated tissue location data were unavailable (Fig. 4a). Analysis of the 299 proteins from HPA which are not present in our proteome shows that 152 of these proteins are validated using RNA data alone. Of these 152 proteins, 35% have been additionally assessed with IHC but have not detected protein expression. In common with HPA, PaxDB and Bliss, we identified the fewest proteins in the ECM (Fig. 4b) however; our MSP does contain 36.4% of the ECM proteins identified by the Matrisome.DB, higher than any other existing proteome. Only 17% of our proteins have been evidenced by MS alone and, unique to our database, 2011 of our proteins have been dual evidenced by both MS and immuno-based techniques (Fig. 4c).

**Figure 5:**
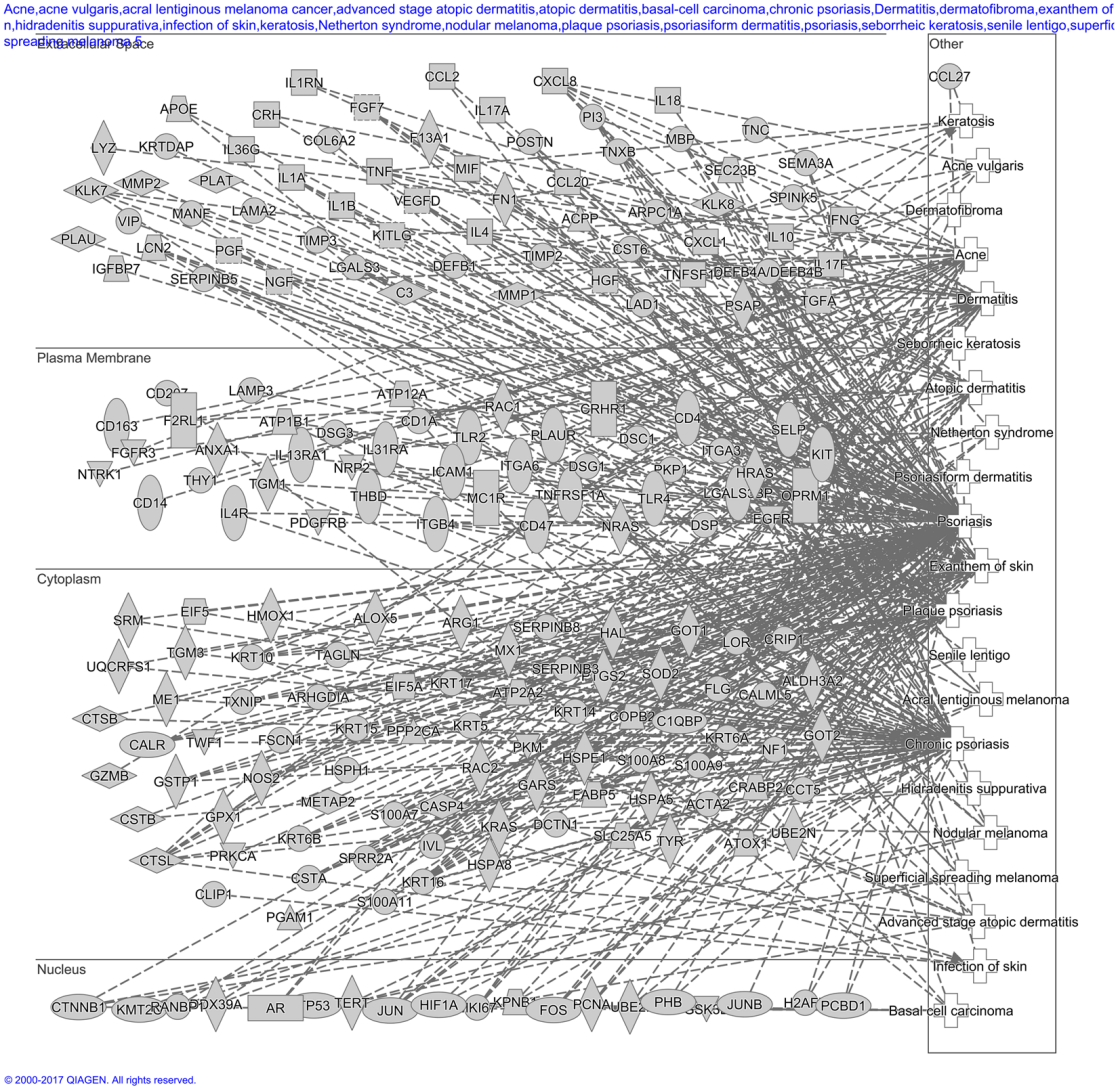
Ingenuity Skin Disease Network of MSP: A detailed breakdown of the gene hits in the skin disease pathways, their associations with each other and subcellular location. The skin diseases included in these pathways are: psoriasis, chronic psoriasis, exanthem of skin, plaque psoriasis, Acne, Dermatitis, advanced stage atopic dermatitis, atopic dermatitis, Netherton syndrome, acne vulgaris, nodular melanoma, keratosis, infection of skin, superficial spreading melanoma, basal-cell carcinoma, hidradenitis suppurativa, senile lentigo, psoriasiform dermatitis, acral lentiginous melanoma cancer, dermatofibroma, seborrheic keratosis. This figure highlights that MSP contains more network connections than the other proteomes assessed (PaxDB and Bliss data in full–Supplementary Fig. 2).

To assess the biological functions associated with the proteins characterised in our MSP we used Ingenuity software to highlight the top level functions and compared these to both Bliss and PaxDB (Fig. 4d). Whilst similar functions are highlighted by Ingenuity pathway analysis for the three largest proteomes, our proteome contains more proteins which can be assigned to top level biological functions (MSP = 45.7%, Bliss = 20.4% and PaxDB = 16.4%). MSP contains: 105 protein hits for hair and skin development and function compared to 37 in Bliss and 57 in PaxDB and; 204 hits for dermatological diseases and conditions compared to 96 in Bliss and 127 in PaxDB, if we focus on the skin specific functions. Thirty seven percent of the top level biological functions including: Cell Cycle; Cell Death and; Survival and Molecular Transport contain no protein hits in either Bliss or PaxDB compared to MSP which had protein hits in all categories. This shows that our MSP offers a more encompassing description of functionality than any other available proteome, offering a more complete profile of protein function. In addition we investigated the Skin diseases pathway in more detail (Fig. 5), this analysis indicates that the MSP contains far more gene hits in the extracellular space than either PaxDB or Bliss (MSP = 60, PaxDB = 26, Bliss = 18 proteins) again indicating that MS analysis alone will lead to incomplete proteomic analysis (Supplementary Fig. 2).

## Disscussion

We have developed a novel approach for defining biological system’s proteomes (www.manchesterproteome.manchester.ac.uk) using a systematic literature-styled review and then applied this method to define the human skin proteome. The MSP is larger than any single MS study and the skin-enriched proteins listed on HPA. Whilst PaxDB does contain more entries in its skin proteome than our database some of these studies include data from cell culture/bodily fluids, which were excluded from our healthy human skin proteome^4,25^. An additional issue with the data from PaxDB is the inability to be able to access the primary sources and therefore any data on experimental protocol or sample preparation. The majority of the data from PaxDB has been collated from ProteomicsDB^26^, again whilst this is an excellent tool for the collation of MS studies to provide data on the entire human proteome, pulling out the more specific data on tissue/organ specific proteomes is very difficult. HPA doesn’t claim to be a proteomics database, but we have used it as a comparison tool as it is one of the largest repositories of data on human proteins in skin. HPA primarily uses RNA data which is sometimes validated at the protein level; however there is no way of easily establishing which entries are validated by IHC. Some of the entries which are validated using IHC use diseased tissue and some also contradictory annotations on whether the protein is detected or not.

Unlike any other proteome or database the MSP identifies 2011 proteins which are dual evidenced using both MS and immune-based techniques, over 80% of which are from multiple studies, giving more confidence of protein presence within the tissue. We have evidenced an additional 437 proteins not present in any of the analysed proteomes or databases; and of these 78% were only evidenced with immune-based techniques, indicating that using MS analysis alone will lead to an incomplete characterisation of the proteome. The ingenuity analysis highlights that our proteome not only contains more hits in all of the top level Kegg pathways, but also contains more hits in the skin specific pathways such as: dermatological diseases and conditions and; hair and skin development and function. This analysis highlights the increased efficacy in our novel methodology by providing a more general description of functionality than any other proteome. A key strength of our methods is the very strict inclusion criteria whereby only healthy human skin was used to evidence protein presence, so any protein from cells, diseased tissue, organ-models or animal tissue were excluded from the search. Here we collate evidence from multiple studies, using multiple samples and techniques which help to reduce false positive results from study and/or sample size bias.

A key advantage of the MSP is the transparency of our data, as within the database is a list of each primary source used to evidence protein presence. Whilst HPA does list the terms ‘healthy human skin’ from three samples, no other information is available with regards to the origin of samples used to gather RNA data, and in addition the IHC analysis is not always completed using healthy skin^3,27^. PaxDB however, does not clearly state how the information about tissue specific proteomes has been collated or where the tissue has been sourced^4,25^. Following the initial application of the methodology we conducted an additional update of our database. The importance of this procedure is highlighted in our results where an additional 1,133 proteins, the majority arising from a single MS study^5^, were added to the MSP. With a growing number of scientific papers being published each year^28^ we wanted to show proof of concept for updating the MSP. One limitation of our methodology is the text mining aspect of the search; to narrow the number of positive papers identified, the protein name must be listed exactly as it is named in Uniprot (given that within the search it is specified in quotation marks). Meaning that any proteins which are commonly abbreviated, such as the late cornified envelope proteins (LCE) and Protein Wnt (WNT) are not present in the list. In addition the LCE proteins have a very limited number of antibodies available for IHC analysis and they also do not appear in any of the MS studies we have analysed, or the entries in PaxDB. The time taken in the manual review process is also a limitation, but does make the data included robust. These methods could be improved in future searches by performing a baseline quality assessment of article for the automatic processes before the manual review, decreasing the work load. In addition more robust protein name/phrase establishment including text processing and lookup expansion would add more information that may have been missed.

In conclusion we have developed, to our knowledge, the first extensive description of a healthy human skin proteome, established using a novel systematic-styled literature review method. This data is stored on a public platform to be used in the scientific community where users can also contribute novel entries. We highlight 437 proteins unlisted in any existing database or comprehensive MS study, in addition to being the only database to dual evidence protein presence. The MSP is up to date and can add new proteins and techniques following scientific advancement. This resource has the potential to aid the development of novel therapeutics for the maintenance of skin health, by acting as a baseline reference. In addition we have developed methodology which can be readily applied to other organs, cell-lines, species or disease-tissues to develop proteome databases for use throughout the scientific community.

## Methodology

### Characterisation of existing skin proteomes/databases

Data were downloaded from Pax.DB (http://pax-db.org/), HPA (http://www.proteinatlas.org) for the skin proteome entries and from Bliss 2016^5^ and proteins were mapped from their Ensembl IDs to UniProtKB entries using the Uniprot Retrieve/ID mapping tool (http://www.uniprot.org/uploadlists/). We ensured all protein IDs were up-to-date and excluded any un-reviewed or non-human protein entries from our collated data. These data were then assessed for common entries between the proteomes/databases. In addition we compared data available from Matrisome.DB (http://matrisomeproject.mit.edu/ecm-atlas/) to assess the percentage of ECM proteins contained in each proteome given the difficulties associated with ECM extraction.

Python script was used to assess each protein’s subcellular location using gene ontology (GO) terms listed on Uniprot. The following rules were applied: if ‘GO:0044420’, ‘GO:0031012’ or ‘GO:0005578’ was listed the protein was classified as ECM, further cross-validation with Matrisome.DB was also performed and if the entry was not part of the core matrisome it was then classified as Extracellular; if ‘GO:0005576’ or ‘GO:0044421’ were listed the proteins were classified as Extracellular and; if ‘GO:0022627’, ‘GO:0016363’, ‘GO:0005882’ or no classification was listen these were classified as Intracellular. The top biological functions were analysed using Gene Enrichment analysis (http://geneontology.org/page/go-enrichment-analysis) and the Quad Venn diagram (Fig. 3) was generated using R script.

### Developing a bioinformatic and systematic literature review approach to define tissue proteomes

Systematic review is a rigorous method often implemented in new policy to influence best practise and is widely used in the medical community to perform meta-analysis of clinical trials evidence^24^. The methodology implements a strict set of inclusion criteria as to what literature can be included in the review, agreed on by a panel^29^. The search terms and inclusion/exclusion criteria of our methodologies have been discussed and agreed by S.A. Hibbert, M. Ozols, M.J. Sherratt, R.E.B. Watson, K.T. Mellody and A. Eckersley and; externally validated by E.C. Bundy. An overview of this systematic methodology and review process is summarised in Fig. 2.

The first step was the identification of original articles whose content focused on healthy human skin. We performed an initial search on the Web of Science, the largest database of available literature (http://wokinfo.com/media/pdf/CCCFactsheet_1-08.pdf), using the search terms: “skin OR cutaneous OR derm* OR epiderm*” AND “human” AND “protein” OR “protemo*” OR “deposit*” OR “express*” OR “synthesis*” using the topic category (searched in the title, abstract and/or keywords). The * ensures all variations of the word endings are included within the search for example derm* would encompass dermal and dermis.

In the first instance we confined the search to original articles written in English only but did not place constraints on publication date (1900–2016). This search however, yielded 456,488 articles which were deemed too many to assess in a manual review process.

To narrow the search we adopted a new strategy, where “protein” OR “protemo*” OR “deposit*” OR “express*” OR “synthesis*” line of query was replaced with the 20,203 review human protein listed on Uniprot. This list of protein names was downloaded from Uniprot (Swiss-prot) in addition to the name of collagen types (Uniprot lists the constituent alpha chains only). Web of Science was queried using batches of 300 protein names, e.g. “14-3-3 protein beta/alpha” OR “14-3-3 protein epsilon” for each of the 20,203 proteins and for “Collagen I/V/X*” OR “Type I/V/X* Collagen” OR “Collagen type I/V/X*”. In addition the previously agreed terms “skin OR cutaneous OR derm* OR epiderm*” AND “human” were also included in this search, and the scope of the search narrowed to English articles published since the year 2000 within the dermatology and biochemistry and molecular biology categories. In addition NOT “Epidermal growth factor” was added to the search to remove false positives which had been identified as an issue in the first search performed. These amendments to the search strategy resulted in 72,230 papers (as of 27.03.15) (Fig. 2a–d).

Following each batch search, to identify literature on the Web of Science, all of the abstracts were downloaded as plain text files, with fifty abstracts in each file. These abstracts were then processed using a Perl algorithm written by M. Ozols (available at: https://github.com/maxozo/Systematic-Abstract-Review.git) before being extracted and saved. The algorithm loaded each abstract individually and performed pre-processing as follows; the 20,203 review Uniprot proteins in addition to ‘skin’, ‘immunohistochem*’ and ‘Mass spec*’ were searched against the 72,230 downloaded abstracts (Fig. 2e). If the abstract contained a protein and/or the additional terms the algorithm output each unique digital object identifier (DOI) into a spreadsheet (see Supplementary Fig. S1) listing: protein names, skin, immunohistochem* and/or mass spec* against the associated DOI to aid the manual review process yielding 20,959 papers (Fig. 2f).

Any paper containing a protein name and skin was manually assessed for experimental evidence of protein presence (all performed by SAH a postdoctoral research associate). The inclusion criteria set for evidence of protein were any methodologies that confirmed protein presence (IHC/immunofluorescence, Western blotting, ELISA, MS and zymography) from healthy, human skin. Excluded were any methodologies that only characterised transcription (mRNA microarrays, PCR, *in situ* hybridisation, miRNA *etc*.) and experimental evidence taken from the following samples: human cells/tissue models; animal cells/tissues or; “unaffected” sites from patients with an underlying condition (Fig. 2g). Data was collected and stored in a Microsoft Access database. Protein names were searched on Uniprot to ensure they were reviewed and the databases’ unique key was assigned to Uniprot protein name and accession number to prevent duplicate data entries being created; Uniprot gene name was also noted. For complete transparency the papers DOIs were documented in addition to: experimental evidence, protein location, skin site, age, sex and any associated disease/mutations if any/all of the information was available. In addition IHC data were also collected from HPA, given that it is the largest publically available deposit of stained tissue. Each protein validated from the initial literature review was searched in HPA and manually reviewed for IHC staining in normal (excluding anal and vulval), healthy, human skin.

As the scientific literature is continuously expanding we conducted an update using the same methodology, for papers since the initial review were searched (31.10.2016). This additional search yielded an extra 1,282 papers for the manual review process. For continual updates a service has been added on the MSP website where users can submit evidence for additional proteins entries. Theses submissions will be reviewed (S. Hibbert/M. Ozols) and if they agree with the set inclusion criteria will be added to our Access and SQL databases and will be visible on our website.

The Venn diagrams used to analyse these data were produced using eulerAPE^30^.

### Ingenuity Pathway analysis

In order to characterise the functionality of MSP, Bliss and PaxDB we uploaded the gene list of each proteome into QIAGEN’s Ingenuity Pathway Analysis (IPA, QIAGEN Redwood City, www.quiagen.com/ingenuity). The core analysis function was used for each of these proteomes, using expression analysis. Network interactions were set on 140 for the molecules per networks and 25 for networks per analysis. We used all node types and all data sources and set the confidence as experimentally observed (no predictions). In addition we set the species as human and tissues and cell lines only dermis, epidermis and skin were selected. The disease and functionality information for gene hits was downloaded in.txt format from the Tree View section and then posthoc analysis was performed on each category to count the number of gene hits in the top level function, duplicate genes were deleted to remove replicate data. This count was then standardised as a percentage of the total number of proteins available in each database (PaxDB, Bliss and MSP). The Network analysis was performed on Dermatological Disease only by selecting these catelgories and exploring the network pathways for MSP, Bliss and PaxDB, these were then sorted by subcellular location to produce the network pathway diagrams (Fig. 5 and Supplementary Fig. 2).

## Electronic supplementary material

Supplementary Information 

Supplementary Data 
