# Supplementary material for: Defining tissue proteomes by systematic literature review
Source: Sci Rep. 2018 Jan 11;8:546. doi: 10.1038/s41598-017-18699-8 (PMC5765030; doi:10.1038/s41598-017-18699-8)
Supplement: Supplementary file 1 — Supplementary Information [file 41598_2017_18699_MOESM1_ESM.doc]

**Defining tissue proteomes by systematic literature review**

**Supplementary Figures**

*Sarah A Hibbert^1#^*, Matiss Ozols^1#^, Christopher EM Griffiths^2, 3,4^, Rachel EB Watson ^2,3,4^, Mike Bell^5^ and Michael J Sherratt^1^**

^1^Division of Cell Matrix Biology & Regenerative Medicine, ^2^Centre for Dermatology Research, Faculty of Biology, Medicine and Health, The University of Manchester, ^3^Salford Royal NHS Foundation Trust, Manchester Academic Health Science Centre, Manchester, UK. ^4^NIHR Manchester Biomedical Research Centre, [Central Manchester University Hospitals NHS Foundation Trust](http://www.cmft.nhs.uk/), Manchester Academic Health Science Centre, UK and ^5^Walgreens Boots Alliance, Thane Road, Nottingham, UK.

^#^Joint first authors.

*Corresponding Authors: Dr Sarah A Hibbert and Dr Michael J Sherratt, 1.528/1.529 Stopford Building, The University of Manchester, Oxford Rd, Manchester, M13 9PT, UK. Telephone: +44 (0)161 275 1439; Fax: +44 (0)161 275 5171; email: [sarah.hibbert@manchester.ac.uk](mailto:sarah.hibbert@manchester.ac.uk), michael.sherratt@manchester.ac.uk

***Supplementary Figure 1: Perl Algorithm output files:*** *A copy of the output from the Perl algorithm that assesses keywords and protein names from the abstract text and their associated DOI. This list was used in the manual reviewing process to identify potential papers to include in the MSP. Red text indicates checked papers that were excluded and green were papers included in the proteome database.*


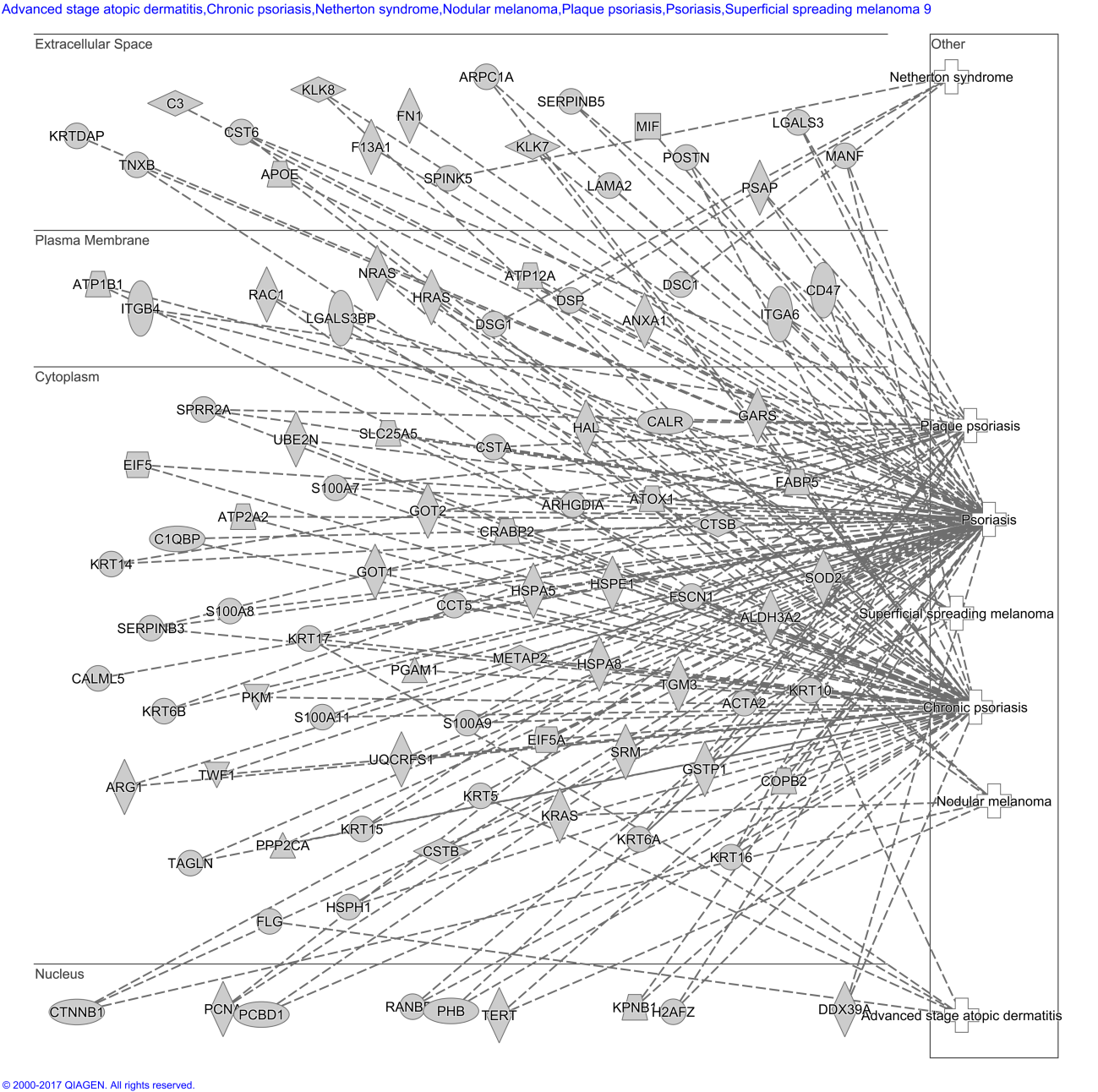
Bliss Data


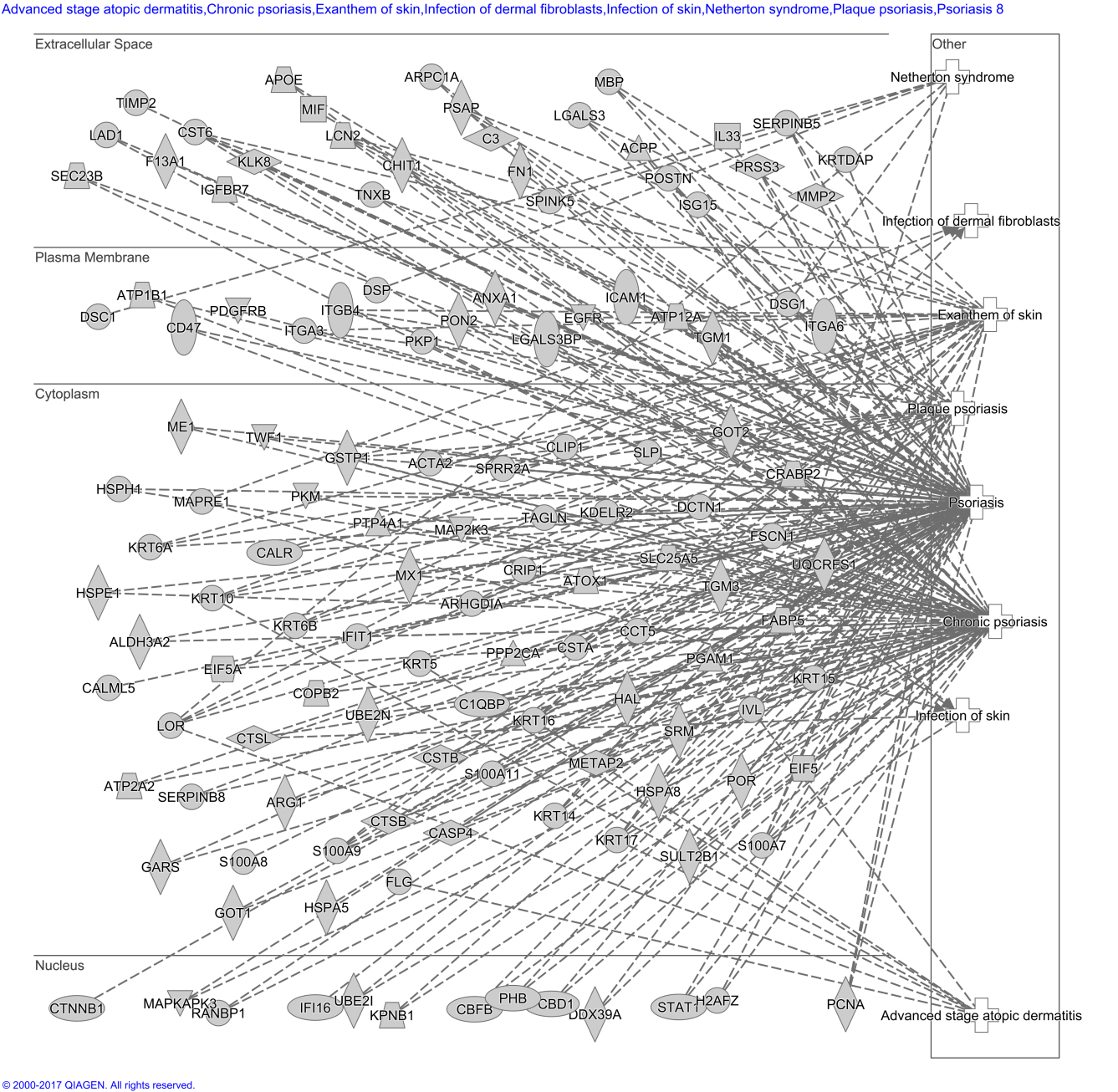
PaxDB

***Supplementary Figure 2: Ingenuity Skin Disease Pathways for Pax.DB and Bliss:*** *The comparison breakdown profiles of Pax.DB and Bliss of the gene hits in the skin disease pathways, their associations with each other and subcellular location. The skin diseases included in these pathways are: psoriasis, chronic psoriasis, exanthem of skin, plaque psoriasis, Acne, Dermatitis, advanced stage atopic dermatitis, atopic dermatitis, Netherton syndrome, acne vulgaris, nodular melanoma, keratosis, infection of skin, superficial spreading melanoma, basal-cell carcinoma, hidradenitis suppurativa, senile lentigo, psoriasiform dermatitis, acral lentiginous melanoma cancer, dermatofibroma, seborrheic keratosis.*
